# Phthalates in Prescription Drugs: Some Medications Deliver High Doses

**Published:** 2009-02

**Authors:** Kellyn S. Betts

Until recently, most of the concern surrounding the health risks of phthalates has focused on the use of these plasticizers in toys, personal care products, food packaging, and medical equipment such as intravenous tubing. A case report in 2004 raised the possibility that certain prescription medications may also be a source of phthalate exposure for some people **[*EHP*** **112:751–753 (2004)]**. That finding prompted a systematic investigation that links phthalate-containing medications with high internal exposure to these chemicals **[*EHP*** **117:185–189; Hernández-Díaz et al.]**.

The 2004 case study pinpointed Asacol®, a medication for treating ulcerative colitis, as a probable source of phthalate exposure. Asacol is covered with an enteric coating of dibutyl phthalate (DBP) that prevents the medication from degrading before it reaches the small intestine. Concentrations of the main metabolite of DBP in the urine of the case study subject corresponded to an uptake of DBP exceeding by two orders of magnitude the 95th percentile reported by the Centers for Disease Control and Prevention in the general population. The concentrations also surpassed the reference dose established for DBP by the U.S. Environmental Protection Agency (EPA) on the basis of animal testing.

To assess possible links between phthalate-containing prescription medication usage and excreted metabolites, the investigators searched National Health and Nutrition Examination Survey (NHANES) data from survey periods between 1999 and 2004 when urine samples were tested for phthalate metabolites and participants were asked about their use of prescription medications. Various enteric-coated medications identified as likely to contain phthalates included mesalamine (the generic form of Asacol), didanosine (an antiretroviral agent), omeprazole (which inhibits gastric acid secretion), and theophylline (used to treat asthma and other lung diseases).

Among the 6 documented mesalamine users, average urine concentrations of DBP metabolites were 50 times higher than those of nonusers. For 2 of the 6 mesalamine users, the DBP metabolite concentrations pointed to uptake exceeding the EPA’s reference dose. Users of the other phthalate-containing medications also had significantly higher concentrations of some metabolites than did nonusers, though the gaps between users and nonusers were considerably smaller than for mesalamine. The NHANES data also showed that at least 3 women who reported taking phthalate-containing medications were pregnant.

These findings call for more investigation, the authors write, particularly because some phthalates cross the placenta and cause reproductive and developmental effects in laboratory animals. In one study of male infants, increasing prenatal exposure to background levels of phthalates was associated with a decrease in the distance between the anus and base of the penis, indicating incomplete male reproductive development **[*EHP*** **113:1056–1061 (2005)]**. The authors of the new study write that phthalate-containing medications are among some of the most widely prescribed drugs in the United States, which implies that many people, including pregnant women, may be exposed to high concentrations of phthalates.

